# Comparison of the Panther Fusion and BD MAX Group B *Streptococcus* (GBS) Assays for Detection of GBS in Prenatal Screening Specimens

**DOI:** 10.1128/JCM.01034-19

**Published:** 2019-10-23

**Authors:** Gregory J. Berry, Fan Zhang, Ryhana Manji, Stefan Juretschko

**Affiliations:** a Infectious Disease Diagnostics, Northwell Health Laboratories, Little Neck, New York, USA; b Department of Pathology and Laboratory Medicine, Donald and Barbara Zucker School of Medicine at Hofstra/Northwell, East Garden City, New York, USA; Marquette University

**Keywords:** group B *Streptococcus*, vaginal-rectal specimens, Panther Fusion GBS assay, BD MAX GBS assay, NAAT

## Abstract

Streptococcus agalactiae or group B *Streptococcus* (GBS) is the cause of early- and late-onset GBS disease in neonates and can present as septicemia, meningitis, and pneumonia. Our objective was to compare the performance of two FDA-approved nucleic acid amplification tests (NAATs), the Panther Fusion and BD MAX systems, for detection of GBS in vaginal-rectal screening specimens. A total of 510 vaginal-rectal prepartum specimens were tested simultaneously in both NAATs following broth enrichment. Assay agreement was calculated using kappa statistics. Overall agreement between assays was 99.0% (505/510; 95% confidence interval, 0.951 to 0.997; kappa = 0.974). Discordant results were retested with both assays and by standard culture. The assays were also compared for workflow characteristics, including time to first results (TFR), total turnaround time (TAT), number of return visits to load additional specimens, and hands-on time (HoT). Using a standard run size of 60 specimens/day, the Panther Fusion assay had a longer TFR (2.4 versus 2.0 h) but showed a shorter overall TAT for all 60 samples (3.98 versus 7.18 h) due to an increased initial sample loading capacity, and it required less labor (35.0 versus 71.3 s/sample) and fewer return visits for loading additional specimens (0 versus 2). The Panther Fusion system also had a larger sample loading capacity (120 versus 24 samples) and greater 8-h throughput (335 versus 96 samples). In summary, the Panther Fusion GBS assay has clinical performance comparable to that of the BD MAX GBS assay but provides a faster TAT, less HoT, and higher throughput.

## Abstract (precis)

Streptococcus agalactiae or group B *Streptococcus* (GBS) is the cause of early- and late-onset GBS disease in neonates and can present as septicemia, meningitis, and pneumonia. Our objective was to compare the performance of two FDA-approved nucleic acid amplification tests (NAATs), the Panther Fusion and BD MAX systems, for detection of GBS in vaginal-rectal screening specimens. A total of 510 vaginal-rectal prepartum specimens were tested simultaneously in both NAATs following broth enrichment.

## INTRODUCTION

GBS is the leading cause of infection in newborns in the United States (1), with 0.22 early-onset GBS disease (EOD) cases per 1,000 live births in 2016 (2). GBS can be vertically transmitted from a colonized mother to her newborn during labor and delivery (intrapartum) and can result in septicemia, meningitis, or, more rarely, pneumonia in newborns, with EOD symptoms appearing within 7 days of birth and late-onset cases appearing as late as 3 months postdelivery (3).

It is estimated that 10% to 30% of pregnant women in the United States are colonized by GBS (4). The current American College of Obstetricians and Gynecologists (ACOG) guidelines for the prevention of EOD recommend universal antepartum screening of pregnant women for GBS colonization at 36 weeks, 0 days to 37 weeks, 6 days weeks of gestation. GBS screening is followed by intrapartum antibiotic prophylaxis for GBS-positive women unless a prelabor cesarean birth is performed in the setting of intact membranes (1). Although this strategy has led to a dramatic decrease in the incidence of EOD since its initial inception in the 1990s (5), culture is a slow process (requires up to 3 days) with suboptimal sensitivity compared to that of molecular assays (6, 7). Nucleic acid amplification tests (NAATs) for the detection of GBS have the potential to remedy the limitations of GBS culture by offering higher sensitivity and rapid time to results (8). Hence, many laboratories have implemented FDA-approved NAATs for routine GBS screening of pregnant women (9). This study compares the clinical performance and workflow characteristics of two NAATs, the Panther Fusion GBS assay (Hologic, Inc., San Diego, CA) and the BD MAX GBS assay (BD Diagnostics, Franklin Lakes, NJ).

(This work was presented in part as a poster and oral presentation at the 2018 Association for Molecular Pathology Annual Meeting and Expo in San Antonio, Texas.)

## MATERIALS AND METHODS

The analytical sensitivity (limit of detection, or LOD) of each NAAT assay was evaluated using quantified strains of Streptococcus agalactiae serotypes III and V. Serial dilutions were made to represent 10, 30, 100, 300, and 1,000 CFU per ml and tested in replicates of ten. Separate LoD panels were made for each serotype. All GBS serotype panels were prepared at the same time, aliquoted into individual tubes for each of ten replicates at each concentration, stored frozen, and thawed on the day of testing.

A total of 510 vaginal-rectal specimens (flocked swabs) received for GBS screening in our laboratory during May 2018 were processed according to CDC guidelines. The swabs were removed from nonnutritive transport medium (Liquid Stuart’s or Amies) and were transferred to 5 ml of Lim broth (Todd-Hewitt broth with 10 μg/ml colistin and 15 μg/ml nalidixic acid) for enrichment. The inoculated Lim broth was incubated at 37°C for 18 to 24 h. After enrichment, specimens were stored at room temperature (15°C to 30°C) for up to 24 h or refrigerated (2°C to 8°C) for up to 5 days before performing both assays. This storage is within the parameters stated in the FDA-approved package insert for each assay.

Prior to testing with both assays, enriched specimens were stripped of individually identifiable information and assigned study-specific identification numbers. For testing with the Panther Fusion assay, 1 ml of the enriched specimen was transferred into an Aptima specimen transfer tube containing 2.9 ml of specimen transport medium. For testing with the BD MAX assay, 15 μl of the enriched specimen was transferred into a sample preparation reagent tube containing 1.5 ml of the reagent. Both assays were performed and interpreted according to the FDA-approved manufacturer’s instructions.

Samples with an invalid result were repeated. Discordant results were further evaluated by retesting with both assays and subculturing from Lim broth onto Trypticase soy agar with 5% sheep blood and incubated at 37°C in 5% CO_2_ for 48 h. The assays’ threshold cycles (*C_T_*) of positive results were retrieved to aid interpretation of discordant results. The Panther Fusion GBS assay has a *C_T_* cutoff of 40 and the BD MAX GBS assay has a *C_T_* cutoff of 37 to determine positivity. Overall assay agreement was calculated using the kappa statistic and associated 95% confidence interval (CI).

A workflow analysis for both systems was conducted to compare the following parameters: (i) number of return visits to the instrument (to load additional samples), (ii) hands-on time (HoT) for each step and overall, (iii) time to first result (TFR), and (iv) turnaround time to results (i.e., total run time, or TAT). Sixty samples, representing a typical daily run size in our laboratory, were run on both platforms on the same day by the same trained operator. The steps of the procedures were timed by an independent observer using a stopwatch. Labor cost was calculated by multiplying the HoT by an average technologist hourly rate of $40 (including wage and benefits).

## RESULTS

Analytical sensitivity was evaluated using Lim broth spiked with GBS serotype III or V at given concentrations (10, 30, 100, 300, and 1,000 CFU/ml). Each concentration had 10 replicates performed. For GBS serotype III, the bacterial concentration with the highest number of replicates detected as positive was 1,000 CFU/ml (10/10; 100% detection) for the Panther Fusion GBS assay and 1,000 CFU/ml (8/10; 80% detection) for the BD MAX GBS assay (Table 1). For serotype V, the lowest concentration with the highest number of replicates was 300 CFU/ml (10/10; 100% detection) for the Panther Fusion GBS assay and 1,000 CFU/ml (10/10; 100% detection) for the BD MAX GBS assay (Table 1).

**TABLE 1:** Limit of detection

Sample size (CFU/ml)	Detection (no. positive/total no.) of GBS serotype:			
	III		V	
	Panther Fusion	BD MAX	Panther Fusion	BD MAX
1,000	10/10	8/10	10/10	10/10
300	8/10	3/10	10/10	7/10
100	3/10	1/10	7/10	2/10
30	2/10	0/10	2/10	1/10
10	1/10	0/10	0/10	0/10
0	0/10	0/10	0/10	0/10

A total of 510 vaginal-rectal specimens collected prepartum were tested simultaneously in both assays for the presence of GBS after broth enrichment. The BD MAX GBS assay, which was the testing platform performed in our laboratory at the time of this study, interpreted 126/510 (24.7%) specimens as GBS positive, and the Panther Fusion interpreted 124/510 (24.3%) specimens as positive. The Panther Fusion GBS assay produced a valid result for 510/510 (100%) of specimens tested, and the BD MAX assay produced a valid result for 504/510 (98.8%) of specimens tested, with 6/510 (1.2%) specimens initially resulting as indeterminate/invalid. Upon a single repeat test on the BD MAX performed immediately after the initial indeterminate/invalid result, all 6 specimens generated a valid result (4 positive, 2 negative) and were included in the final data set for analysis.

Table 2 illustrates the results of the comparison between the Panther Fusion and BD MAX GBS assays. Out of the 510 specimens included in the final data set, there were 5 discordant results between the two assays, demonstrating an overall percent agreement (OPA) of 99.0% (95% CI, 0.951 to 0.997; kappa = 0.974). The positive percent agreement (PPA) and negative percent agreement (NPA) were 96.9% (95% CI, 0.922 to 0.988) and 99.7% (95% CI, 0.985 to 1.0), respectively. Additional testing for the five specimens with discordant results is depicted in Table 3. The four specimens that initially tested positive by the BD MAX GBS assay and negative by the Panther Fusion GBS assay repeated as negative on both assays. In addition, *C_T_* values and amplification curves for the four initial positive results were examined for the BD MAX assay, and no *C_T_* value or discernible amplification was observed upon repeat. The one specimen that initially tested positive by the Panther Fusion GBS assay (*C_T_* = 36.3) and negative by the BD MAX GBS assay was repeated and again was positive by the Panther Fusion GBS assay (*C_T_* = 37) and negative by the BD MAX GBS assay. Culture was also performed for each of five discordant specimens and yielded negative results (Table 3).

**TABLE 2:** Comparison of the Panther Fusion and BD MAX assays for GBS detection

Assay result^*a*^ (*n* = 510)		
Panther Fusion	BD MAX	
	+	−
+	123	1
−	4	382

a PPA (95% CI), 96.9% (0.921–0.991); NPA (95% CI), 99.7% (0.986–1.0); OPA (95% CI), 99.0% (0.977–0.997).

**TABLE 3:** Results of additional testing performed on discordant specimens^*a*^

Specimen	Initial result		Repeat result		Culture result
	BD MAX	Panther Fusion	BD MAX	Panther Fusion	
1	+/*C_T_* = 35	−	−	−	Isolate not recovered
2	+/No *C_T_*	−	−	−	Isolate not recovered
3	+/No *C_T_*	−	−	−	Isolate not recovered
4	+/No *C_T_*	−	−	−	Isolate not recovered
5	−	+/*C_T_* = 36.3	−	+/*C_T_* = 37	Isolate not recovered

a *C_T_*, cycle threshold.

Northwell Health Laboratories processes more than 1,300 specimens per month on average for GBS testing, necessitating consideration of workflow efficiency in any platform decision. Both assays and systems were evaluated for various workflow parameters and compared for testing of 60 specimens, the highest daily volume our laboratory typically encountered at the time of this study. Based on instrument loading capacity, 60 specimens represented 3 testing batches for the BD MAX system (24-specimen capacity), while the Panther Fusion was able to be loaded as a single testing batch (120-specimen capacity). Although the BD MAX has an earlier time to first results, finishing the first 24 specimens in 2.0 h compared to the Panther Fusion finishing the first 5 specimens in 2.4 h (with 5 additional results every 5 min), the Panther Fusion assay performed with a faster overall TAT (3.98 versus 7.18 h) for all 60 samples, had less hands-on time (0.58 versus 1.19 h), and required less preparation time (35.0 versus 71.3 s) per sample. No additional loading of specimens was necessary when operating the Panther Fusion platform, but when using the BD MAX assay, 2 return visits to load additional specimens were needed. This difference was due to the larger sample loading capacity (120 versus 24 samples) of the Panther Fusion system, which allowed all 60 specimens in the workflow analysis to be loaded in a single run and only requiring a single initial visit to load all samples and reagents. In contrast, the BD MAX system required three separate batches to accommodate all 60 specimens, resulting in 2 return sample loading visits: one visit at the end of batch 1 to load batch 2 and one visit at the end of batch 2 to load batch 3. The shorter labor time achieved by the Panther Fusion system (35.0 versus 71.2 s/sample) can amount to a savings of $21,699 per year, assuming an average of 16,000 samples per year are received in a laboratory solely for GBS testing at an estimated salary of $40/h for a laboratory medical technologist.

## DISCUSSION

Demand for more sensitive testing with faster turnaround times is constantly increasing, while at the same time, many laboratories are experiencing increasing testing volume and workforce shortages. Due to these demands, testing platforms that allow laboratories to partially (or fully) automate testing and deliver these results are becoming necessary. In this study, we evaluated the performance of two such platforms, BD MAX and Panther Fusion, for the detection of GBS in prenatal screening specimens.

Overall, Panther Fusion had a slightly lower LOD than BD MAX, and both assays showed similar analytical performance, exhibiting a high PPA (96.9%), NPA (99.7%), and OPA (99%). Analysis of the five discordant results obtained in the study showed that BD MAX could not replicate the initial positive results obtained on four of the specimens, while Panther Fusion consistently called these results negative in both the initial and repeat testing results. Culture also yielded negative results in each of these four cases. Conversely, Panther Fusion interpreted one sample as positive, while BD MAX interpreted this same specimen as negative. Repeat results were identical to initial results for both assays (Panther Fusion^+^/BD MAX^−^). Culture results for the same specimen were negative. While culture was negative, it is quite possible that this specimen was still GBS positive, especially considering that culture has been shown to be less sensitive than various molecular methods for the detection of GBS (6, 7). It is also possible that Panther Fusion detected GBS in this specimen due to the assay’s lower LOD for GBS than that of BD MAX, as shown in Table 1. Differences in the Lim broth inoculum used in the assays (15 μl of the enriched specimen in the BD MAX GBS assay versus 1 ml of specimen for the Panther Fusion GBS assay) also could contribute to the analytical differences seen between the two assays.

While a modest difference in analytical performance was observed between these two assays, there was a contrast when workflow in a high-volume testing laboratory setting was considered. In this respect, Panther Fusion outperformed BD MAX in overall sample loading capacity, HoT, and overall TAT. The maximum number of samples per instrument per 8-h shift (throughput) for the Panther Fusion GBS assay is 335 samples and for the BD MAX GBS assay is 96 samples. This implies that the Panther Fusion system has the capability of processing 3.5 times more samples in an 8-h shift than the BD MAX instrument. One important point to keep in mind when considering the workflow analysis results is that if laboratory GBS screening volumes are significantly less than 60 specimens a day (especially less than 24 specimens, the BD MAX maximum batch size), the performance difference between the two systems would be less pronounced.

While molecular testing for GBS screening has increased sensitivity compared to that of culture, one important caveat is that it does not provide an isolate for antimicrobial susceptibility testing. This type of testing is required for penicillin-allergic patients and must be available. This should be taken into account when considering a switch to molecular methods for GBS screening.

Overall, this is the first study comparing the Panther Fusion and BD MAX GBS assays for clinical performance and workflow. The Panther Fusion GBS assay showed clinical performance comparable to that of the BD MAX GBS assay but exhibited a superior workflow, including a less labor-intensive procedure, faster turnaround to results, and greater sample throughput, all of which could reduce operating costs.
